# Resveratrol modulates FABP5 to reduce neuronal apoptosis following ischemic stroke

**DOI:** 10.1515/biol-2025-1253

**Published:** 2026-01-20

**Authors:** BingFeng Xing, Xin Zhou, Min Hong, WeiHao Lin, Changqin Xiang

**Affiliations:** The First Affiliated Hospital/The First Clinical Medicine School of Guangdong Pharmaceutical University, Guangzhou, Guangdong, China; The First Affiliated Hospital, Sun Yat-sen University, Guangzhou, China

**Keywords:** FABP5, fatty acid–binding protein, ischemic stroke, neuronal apoptosis, resveratrol

## Abstract

Fatty acid–binding proteins (FABPs) influence cellular energy metabolism by regulating fatty acid kinetics. They also play a vital role in neuronal apoptosis following cerebral infarction. Resveratrol (RSV) has demonstrated neuroprotective effects in ischemic stroke; however, its regulatory impact on FABPs and associated pathways requires further investigation. This study aimed to explore the potential mechanisms by which RSV protects ischemic stroke neurons by regulating fatty acid metabolism. A weighted gene co-expression network analysis revealed significant enrichment of FABP5 in fatty acid metabolism–related pathways in rats with middle cerebral artery occlusion (MCAO). Modulating FABP5 expression level may influence post-infarction neuronal recovery. Molecular docking experiments demonstrated that RSV exhibited strong binding affinity with FABP5. In the MCAO-group of rats, administering different doses of RSV led to a significant decrease in cerebral infarct area and improved neurological function with increased RSV doses. Concurrently, the expression of FABP5 and neuron-specific enolase in brain tissue decreased, whereas the expression of the brain-derived neurotrophic factor increased and neuronal morphology improved. Further experiments using FABP5 overexpression and inhibition models revealed that FABP5 overexpression exacerbated neuronal apoptosis and suppressed the expression of adenosine monophosphate (AMP)-activated protein kinase (AMPK) protein, whereas FABP5 inhibition reduced neuronal apoptosis and enhanced AMPK protein expression. RSV downregulates FABP5 expression in cerebral infarction tissues and potentially mediates the AMPK-related pathways to ameliorate neuronal apoptosis.

## 1 Introduction

Ischemic stroke (IS) is an acute neurological disorder caused by the interruption of cerebral blood flow due to thrombosis or embolism. It is the second leading cause of death globally. According to statistics, IS causes approximately 5.9 million deaths and 102 million cases of disability annually, with an incidence rate of 45 per 100,000 people in 2019 [1]. During the onset of IS, the obstruction of blood flow through the carotid and vertebral arteries leads to significant brain damage and neurological deficits [2]. Free fatty acids (FAs) in neuronal cells are crucial for both signal sensing and transduction. FA signaling pathways and their associated proteins related to neuronal protection and repair in IS have garnered increasing attention in the scientific community in recent years. In the cytoplasm, free FAs are transported to specific subcellular structures and metabolic pathways by FA-binding proteins (FABPs) [3]. FABPs are important protein markers in FA metabolic pathways related to neuronal injury and repair in IS. They regulate mitochondrial energy metabolism, oxidative stress responses, and blood–brain barrier damage in the brain [4], 5]. Previous studies have confirmed that FABPs are intracellular proteins that bind and transport FAs, exacerbating central nervous system (CNS) cell injury by inhibiting cellular energy supply and downstream lipid oxidation and stress responses [6], 7]. Among the FABP subtypes, FABP3, FABP5, and FABP7 have received considerable attention in IS research [8]. FABPs are widely researched as biomarkers of IS [9], [10], [11].

Resveratrol (RSV) is a natural polyphenolic compound commonly found in medicinal plants. It has been shown to exert various beneficial effects, including neuroprotection, cognitive improvement, and immune regulation [12], 13]. Recent meta-analyses indicate that RSV can effectively reduce cerebral ischemic infarction, edema volume, and blood–brain barrier damage, while also improving neurological function. These findings suggest that RSV may serve as a promising neuroprotective agent following cerebral infarction [14]. However, the neuroprotective mechanisms of RSV have not yet been fully elucidated. RSV also plays a significant role in regulating FA metabolism. RSV can modulate the lipid order and organization of the cytoplasmic membrane, which is closely related to critical cellular functions such as membrane sorting and transport [15]. The neuroprotective effects of RSV in IS neurons require further investigation to determine whether they are mediated by the modulation of FABPs.

In this study, we used a middle cerebral artery occlusion (MCAO) model and employed bioinformatics analysis to identify FABP subtypes associated with neuronal repair following cerebral infarction. RSV was administered through oral gavage to the MCAO model rats. The changes in IS neuronal cells were observed, and the effects of FABP overexpression and inhibition on key proteins involved in FA metabolism were investigated. Furthermore, their relationship with neuronal injury was explored. This study was conducted to understand the potential mechanisms by which RSV protects IS neurons by regulating FA metabolism.

## 2 Materials and methods

### 2.1 Animals and design

A total of 64 specific-pathogen-free male Sprague–Dawley (SD) rats (180–200 g) were purchased from Zhuhai Bestest Biotechnology Co., Ltd. All rats were housed individually in transparent cages for 7 days under a 12-h light/12-h dark cycle (08:00–20:00). They had free access to water and food, and the room temperature was maintained at approximately 25 °C. Before the experiment, the rats were fasted for 6 h with free access to water.

This study comprised two parts: biosignal analysis and animal experiments. The animal experiment was further divided into two subparts. In part 1, all rats were selected and divided into five groups: sham surgery, MCAO, RSV5, RSV10, and RSV20. The rats in the RSV5, RSV10, and RSV20 groups were administered RSV at doses of 5 mg/kg, 10 mg/kg, and 20 mg/kg, respectively, through oral gavage. In part 2, the experimental rats were divided into three groups: MCAO, FABP5 overexpression, and FABP5 inhibition. The rats in both the FABP5 overexpression and FABP5 inhibition groups were administered RSV at a dose of 10 mg/kg through oral gavage. After 1 week of acclimatization, the rats were randomly divided into a sham surgery group (*n* = 8) and an MCAO model group (*n* = 56). The MCAO model group was further subdivided into MCAO, RSV5, RSV10, RSV20, FABP5 overexpression, and FABP5 inhibition groups.

**Ethical approval:** The research related to animal use has been complied with all the relevant national regulations and institutional policies for the care and use of animals, and has been approved by the Ethics Committee of the First Affiliated Hospital of Guangdong Pharmaceutical University (Approval No. GYFYDWSY2024029).

### 2.2 MCAO and sham surgery group rat modeling

MCAO group: The rats in the MCAO model group were first anesthetized using an oxygen anesthesia machine (ABS, Yuyan, Shanghai, China) to induce a pain-free state. Then, they were placed in a supine position, and the neck area was shaved and disinfected. The rats were then positioned on a surgical table, and their body temperature was maintained at approximately 37 °C. The head was secured in a holder, and a midline incision was made in the skin of the neck. Layers were dissected to expose the left carotid artery and vagus nerve along the trachea. Upon identifying the “Y”-shaped vascular structure, the external carotid artery and the distal end of the internal carotid artery were ligated using surgical sutures. A 0.15-mm-diameter filament was inserted to induce ischemic occlusion. The filament was removed after 30 min of occlusion, and the incision was sealed using an electric coagulator (HH-V50, Jiangsu, China) and sutured layer by layer. The rats were moved to a warming chamber for a gradual recovery. Throughout the process, necessary medical interventions and monitoring of body temperature, heart rate, respiratory rate, and neurological function were conducted.

Sham surgery group: A total of eight SD rats were used in the sham surgery group. Only a skin incision was made without inducing ischemic injury, and the rats were administered an equivalent volume of saline through oral gavage.

FABP5 overexpression and inhibition groups: The FABP5-PCDH-CMV-EF1A-EGFP-T2A-PURO-overexpression plasmid and the pLKO.1-EGFP-puro-FABP5shRNA-knockdown plasmid were constructed, followed by lentiviral packaging. The viral supernatant was collected and filtered through a 0.45-μm filter, and the lentiviral titer was measured. The corresponding lentiviral particles were then injected into the two groups of rats, labeled as the overexpression (OVER) and the inhibition (SH) groups.

### 2.3 Biosignaling pathway enrichment analysis in the MCAO group rats

The GSE166162 dataset was downloaded from the GEO(Gene Expression Omnibus)database (https://www.ncbi.nlm.nih.gov/geo/). A weighted gene co-expression network analysis (WGCNA) was performed using R language (version 3.6.2) to explore potential biosignaling pathway enrichment in the MCAO group rats. A total of 12 samples from the Affymetrix Rat 230 2.0 array were divided into sham and MCAO groups, with 6 samples in each group (*n* = 6). The GEOquery package was used to obtain the expression matrix, and gene annotation was performed using the GPL1355 platform. An “unsigned” co-expression network was constructed using WGCNA. The correlation between sorted module eigengenes (MEs_col) and trait data (traitData) was calculated using the Pearson method. Genes associated with MCAO expression were screened based on gene significance (GS) > 0.6 and module membership (MM) > 0.6. The STRING database (https://string-db.org/) was used to construct a protein–protein interaction (PPI) network and perform pathway enrichment analysis. A total of 56 genes, including 55 genes screened by WGCNA and 4 genes of interest from previous studies [FABP4, FABP5, neuron-specific enolase (NSE), brain-derived neurotrophic factor (BDNF)], were included in the network. In the STRING database, the species was set to *Rattus norvegicus*, and the Markov Cluster Algorithm (MCL) algorithm was used for cluster analysis. Pathway enrichment analysis of the screened genes was performed using the Gene Ontology and Kyoto Encyclopedia of Genes and Genomes databases. The results were visualized using the ggplot2 package in R.

### 2.4 Molecular docking analysis of RSV and FABP

The active structure of the target protein was obtained from the PDB database (https://www.rcsb.org/) in a PDB file format. The SDF file of the target small molecule, RSV, was retrieved from the PubChem database (https://pubchem.ncbi.nlm.nih.gov/) using the search term “Resveratrol.” Semi-flexible docking was performed using AutoDock 4.2.6. The results showed a low binding energy between the ligand (RSV) and the receptor (FABP), indicating strong binding affinity.

## 3 Effects of RSV on neuronal morphology and function in the MCAO group rats

### 3.1 Grouping and drug administration in the MCAO group rats

After establishing the MCAO model, the rats in the first part of the experiment were numbered 1–32 and those in the second part were numbered 33–56. Random numbers were generated using the “RandBetween (1,24)” function in Excel, and the rats were grouped according to the random sequence. In the first part of the experiment, rats 1–8 were assigned to the MCAO control group, 9–16 to the RSV5 group, 17–24 to the RSV10 group, and 25–32 to the RSV20 group. In the second part of the experiment, rats 33–40 were assigned to the MCAO control group, 41–48 to the FABP5 OVER group, and 49–56 to the FABP5 SH group. Further, the rats in the RSV5, RSV10, and RSV20 groups were administered RSV at doses of 5 mg/kg, 10 mg/kg, and 20 mg/kg, respectively, through oral gavage once daily for seven consecutive days. The rats in the MCAO control group were administered an equivalent volume of saline through oral gavage. Moreover, the rats in both the FABP5 OVER and SH groups were administered RSV at a dose of 10 mg/kg through oral gavage once daily for seven consecutive days. The rats in the MCAO control group were administered an equivalent volume of saline through oral gavage. The rats were observed for 7 days. The animal inclusion criteria were as follows: healthy male SD rats with appropriate body weight and normal behavior. The exclusion criteria were as follows: rats exhibiting abnormal behavior, failing to gain weight, or dying during adaptive feeding, as well as rats that died or failed to develop ischemic symptoms within 24 h after modeling.

### 3.2 Behavioral monitoring of the rats with MCAO in each group

**Longa neurological score:** The neurological deficits were scored as follows: 0 points for no neurological deficits, 1 point for incomplete extension of the left forepaw, 2 points for circling to the left while walking, 3 points for falling to the left (paralyzed side) while walking, and 4 points for inability to walk spontaneously, with loss of consciousness. The neurological scores were assessed on day 0 (before the experiment) and on days 1, 3, 5, and 7 of the experiment.

**Open field test:** The open field test apparatus (SG-ANE04, Sengene, Guangzhou,China)consisted of a black plastic box with dimensions of 90 cm (length) × 90 cm (width) × 45 cm (height). A camera was mounted above the box to capture the entire field of view. Before each test, the floor and walls of the box were wiped with alcohol to eliminate residual odors or excretions from previous animals. The testing began on day 7 of the experiment, with one rat tested at a time for 5 min per session. A minimum interval of 24 h was maintained between tests. A rat was placed at the starting point of the open field, and the recording device was activated to track the animal’s movement trajectory. After data collection, the field was divided into a 3 × 3 grid using analysis software. The total distance traveled (total distance) and the duration of immobility (time the rat stops moving) were recorded and analyzed. Data analysis was performed using ImageJ (V1.0, NIH, USA).

### 3.3 Comparative analysis of cerebral infarct area, neuronal morphology, and related factor expression in the rats with MCAO from each group

**Brain tissue sampling and Western blot (WB) analysis:** The brain tissue was rinsed with 0.9 % saline and placed on an ice platform. The left and right cerebral hemispheres were separated. The left cerebral hemisphere was homogenized. Specifically, the right brain tissue was placed in a centrifuge tube and stored in a freezer at −80 °C. Whole-cell lysis buffer was added, followed by ultrasonic homogenization. The sample was kept on ice for 15 min and then centrifuged (12,000 rpm, 4 °C, 15 min). The supernatant was transferred to a new centrifuge tube. Protein concentrations of BDNF, NSE, FABP3, FABP4, FABP5, and AMP-activated protein kinase (AMPK) were measured using corresponding assay kits. Further, radioimmunoprecipitation assay lysis buffer (ES-8150; Ecotop, China), bicinchoninic acid protein assay kit (EK-5001; Ecotop), polyvinylidene difluoride membrane (ISEQ00010; Millipore, USA), AMPKa1/AMPKa2 mouse mAb (1:1000, A27099, ABclonal, China), BDNF polyclonal (1:1000, 28205-1-AP; Proteintech, China), FABP4 (1:2000, ab92501; Abcam, England), FABP5 (1:1000, ab151772; Abcam, and FABP3) (1:800, A8789; ABclonal) were used in the study.

**TTC staining:** 2,3,5-Triphenyltetrazolium chloride (TTC) staining for infarct area assessment: In this study, a 2 % TTC staining solution was applied to brain tissues on the 7th day post-surgery to evaluate ischemic injury. For this, fresh brain tissue slices were completely immersed in a 2 % TTC staining solution and incubated at 37 °C for 30 min. The color changes were observed during incubation. After staining, the tissues were gently rinsed with phosphate-buffered saline (PBS), and the resulting stained sections were photographed. ImageJ software was used for image recognition and analysis to calculate the percentage of TTC infarct area as follows: Infarct volume ratio = (sum of white infarct areas in all slices)/(Sum of total brain slice areas) × 100 %.

**Hematoxylin and eosin staining:** Morphological changes in stained brain tissue: Paraffin-embedded brain tissue sections were deparaffinized in xylene for 10 min. The sections were then rehydrated by sequentially immersing them in 95 %, 85 %, and 70 % ethanol gradients for 5 min each. After rehydration, the samples were washed three times with PBS, each for 5 min. Hematoxylin staining was performed for 10 min, followed by differentiation using 1 % hydrochloric acid ethanol. Eosin staining was then applied for 3 min. The tissue sections were dehydrated through an ethanol gradient, mounted with neutral resin, and air-dried before being photographed.

### 3.4 Flow cytometry analysis of neuronal apoptosis

The neuronal apoptosis was detected using the Annexin V–fluorescein isothiocyanate (FITC)/propidium iodine (PI) Apoptosis Kit (E-CK-A211, Elabscience Biotechnology, China). In this procedure, apoptosis was induced following the experimental protocol. The cells were centrifuged at 300*g* for 5 min, and the supernatant was discarded. The cell pellet was collected, washed once with PBS, and gently resuspended, followed by cell counting. A total of 1 × 10^6^ resuspended cells were centrifuged at 300*g* for 5 min, and the supernatant was discarded. The cells were washed once with PBS, centrifuged again, and the supernatant was discarded. The cell pellet was resuspended in 100 μL of diluted 1× Annexin V binding buffer. Then, 2.5 μL of Annexin V–FITC reagent and 2.5 μL of PI reagent (50 μg/mL) were added. The mixture was gently mixed and incubated at room temperature in the dark for 20 min. After incubation, 400 μL of diluted 1× Annexin V binding buffer was added, and the sample was mixed thoroughly. Apoptosis was analyzed using a BD FACSCanto II flow cytometer (FACSCanto™ II, Becton, Dickinson and Company, America).

### 3.5 Quantitative reverse transcription–polymerase chain reaction

Primers were designed using the National Center for Biotechnology Information database (https://www.ncbi.nlm.nih.gov/), and specific binding tests were performed using primer BLAST. Rat brain tissue was homogenized at 4 °C using the Wonbio-KS rapid tissue disruptor (WK1, Shanghai, China). Total RNA was extracted with RNAEx ZOL (EK5301; ECOTOP, China) and the FastPure Total RNA Extraction Kit (EK1328; ECOTOP). cDNA was synthesized using the PrimeScript RT Master Mix (RR036A; TaKaRa Bio, Japan), and quantitative polymerase chain reaction (qPCR) was performed with TB Green Premix Ex Taq II (RR820A; TaKaRa Bio). All samples were standardized to the internal reference level, and the reference primers for each gene were as follows:

**Table:** 

Gene	Forward (5′ → 3′)	Reverse (5′ → 3′)
BDNF	TTTGGGGCAGACGAGAAAGC	ACCTGGTGGAACTCAGGGT
NSE	CCAATCGAAGCTCAACCGAAG	TCGTCAGACCTCTCGAACCT
FABP5	AAACTGAGACGGTCTGCACC	TGGGAATCACATCGCGTCTC
AMPK	GAGAACATCAATTGACAGGCCAT	CCCGCATACAGCCTTCCTGA

### 3.6 Statistical analysis

The data were presented as mean ± standard deviation (SD) and analyzed using Prism 8.5 software (V8.5; GraphPad, USA). One-way analysis of variance, followed by Tukey’s honestly significant difference post hoc test, was used for multiple treatment comparisons. A P value < 0.05 indicated a statistically significant difference.

## 4 Results

### 4.1 Close association of IS neuronal injury and protection with FABP5, an FA metabolism pathway protein, with a strong binding affinity to RSV

**Figure 1:**
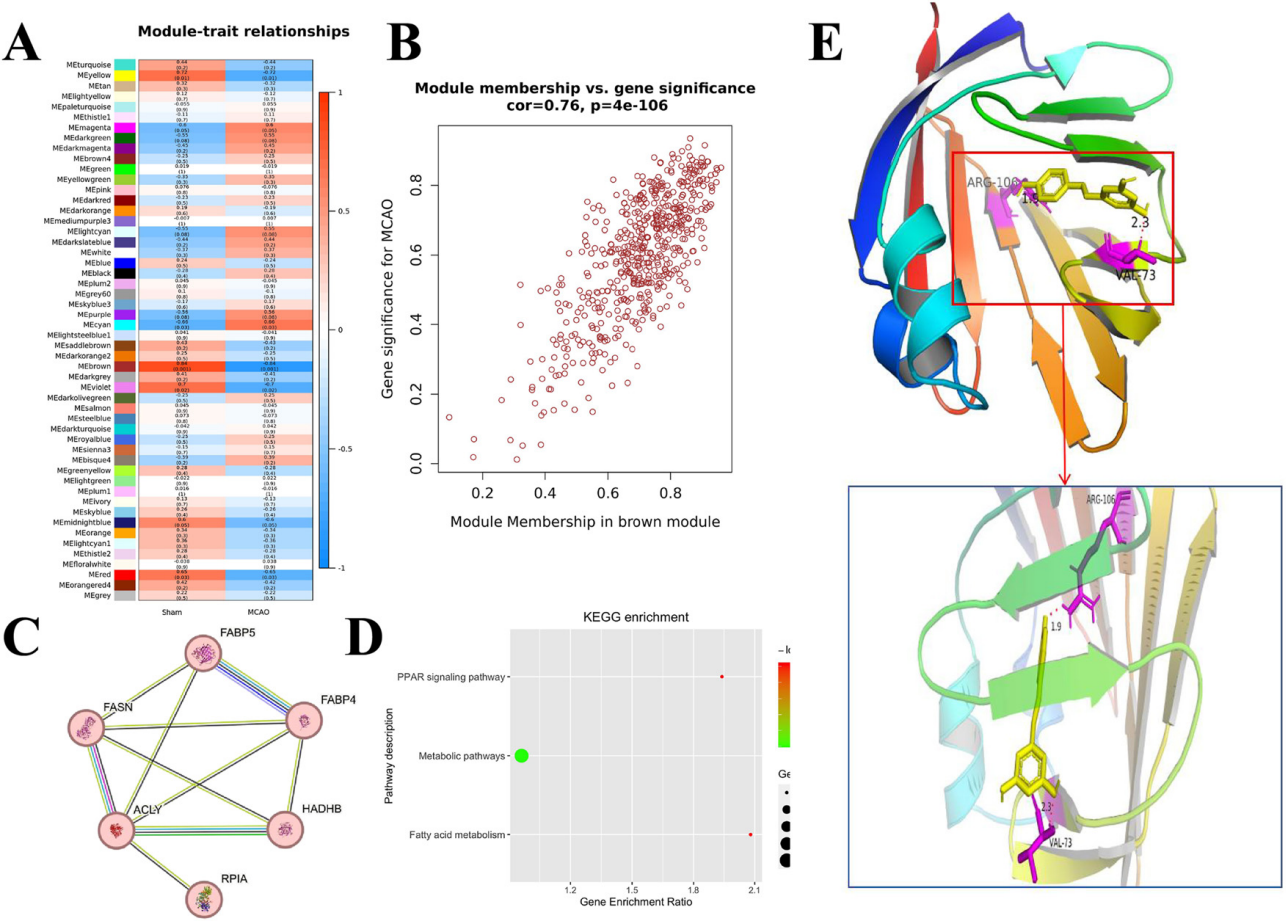
Enrichment analysis of biological signaling pathways in MCAO rats and molecular docking of RSV with FABP. (A) WGCNA analysis of modules and phenotype association. Samples labeled “Sham” were assigned to the sham operation group (Sham), and those labeled “MCAO” were assigned to the model group (MCAO). Module – phenotype association analysis was performed. The MEbrown module, with a negative correlation coefficient of 0.84 (*P* = 0.001), was selected as the module of interest, with a focus on the MCAO group. (B) Gene distribution and GS in the module of interest. MM and GS genes from the MEbrown module in the MCAO group were extracted. Genes highly correlated with the phenotype were screened using the criteria of GS > 0.6 and MM > 0.6. These genes, which are also key genes associated with the phenotype, were visualized. A total of 56 genes were identified to be significantly associated with the “MCAO” phenotype. (C) Construction of the PPI network and identification of the FABP5 core network. A PPI network was constructed using the 56 genes, and the core network centered on FABP5 was identified. (D) pathway enrichment analysis. FABP5’s association with metabolism pathway enrichment analysis demonstrated that FABP5 was primarily involved in metabolic pathways. (E) Semi-flexible molecular docking analysis of resveratrol with FABP5 revealed binding sites between resveratrol and FABP5.

Cluster analysis, soft threshold screening, module construction, phenotype data correlation analysis, and GS analysis of MCAO rat samples revealed that 56 genes significantly associated with the “MCAO” phenotype. The PPI pathway enrichment analysis showed that genes in cluster 1 were primarily related to FA metabolism. This suggested that the regulation of FABP5, an FA metabolism pathway protein, was closely linked to IS neuronal injury and protection (Figure 1A–D). Semi-flexible molecular docking experiments further demonstrated that RSV bound to FABP5 (Figure 1E), indicating an interaction between RSV and FABP5.

### 4.2 Improvement of motor and cognitive functions in rats with MCAO by RSV

**Figure 2:**
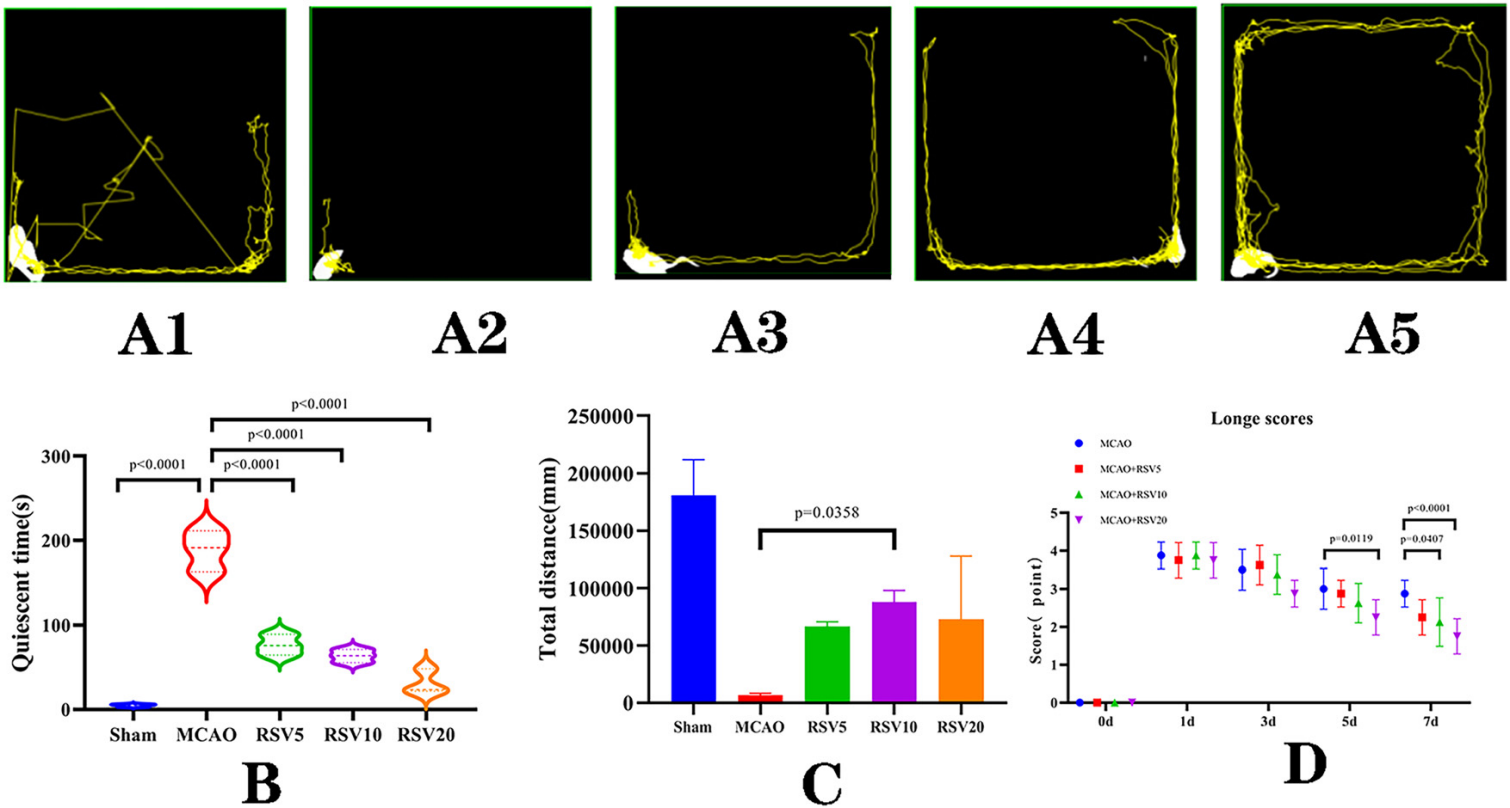
Behavioral monitoring and comparison in MCAO rats. Assessment of motor function in rats. (A) Open field test trajectory diagrams. The yellow central point represents the starting position, and the yellow lines indicate movement trajectories. A1–A5 correspond to the blank control group, MCAO control group, RSV5, RSV10, and RSV20 groups, respectively. (B) Statistical analysis of immobility time in the open field test (unit: s). (C) Statistical analysis of total distance traveled in the open field test (unit: mm). (D) Longa scores for each group of rats.

In the first part of the experiment, the open field test and Longa neurological scores were used to assess the behavioral and neurological outcomes of the rats. The movement trajectories in the open field test showed that the sham-group rats had the longest total distance traveled and the shortest stationary time, and exhibited central grid exploration behavior (Figure 2A1). In contrast, the MCAO-group rats remained stationary in the corners for long periods, had a small range of activity, and moved slowly (Figure 2A2). After RSV treatment, the motor functions of the rats in each group significantly recovered, with increased total distance traveled and reduced stationary time. This was particularly noted in the RSV10 and RSV20 groups. The RSV20-group rats displayed more central exploration behavior compared with the RSV10-group rats (Figure 2A3–A5). The statistical results of stationary time in the open field test showed significant differences between the RSV treatment groups at different doses and the MCAO control group (*P* < 0.001) (Figure 2B). The statistical results of the total distance traveled in the open field test showed a significant difference between the RSV10 and MCAO control groups (*P* = 0.0358) (Figure 2C). The Longa scores of rats in the RSV20 group were significantly different from those in the MCAO group on day 5 (*P* = 0.0119). The RSV10 and RSV20 groups showed significant differences compared with the MCAO group on day 7 (*P* = 0.0407 and *P* < 0.0001, respectively) (Figure 2D). The aforementioned results indicated that the behavioral and cognitive functions of the MCAO-group rats were significantly impaired, but their behavioral abilities were significantly improved after RSV treatment. The improvement in the motor and cognitive behavioral abilities of the MCAO-group rats gradually increased with higher concentrations of RSV. This indicated a significant dose-dependent effect of RSV in improving the behavioral aspects of the MCAO group rats.

### 4.3 Amelioration of neuronal morphology in rats with MCAO by RSV and the positive correlation of the degree of improvement with RSV concentration

**Figure 3:**
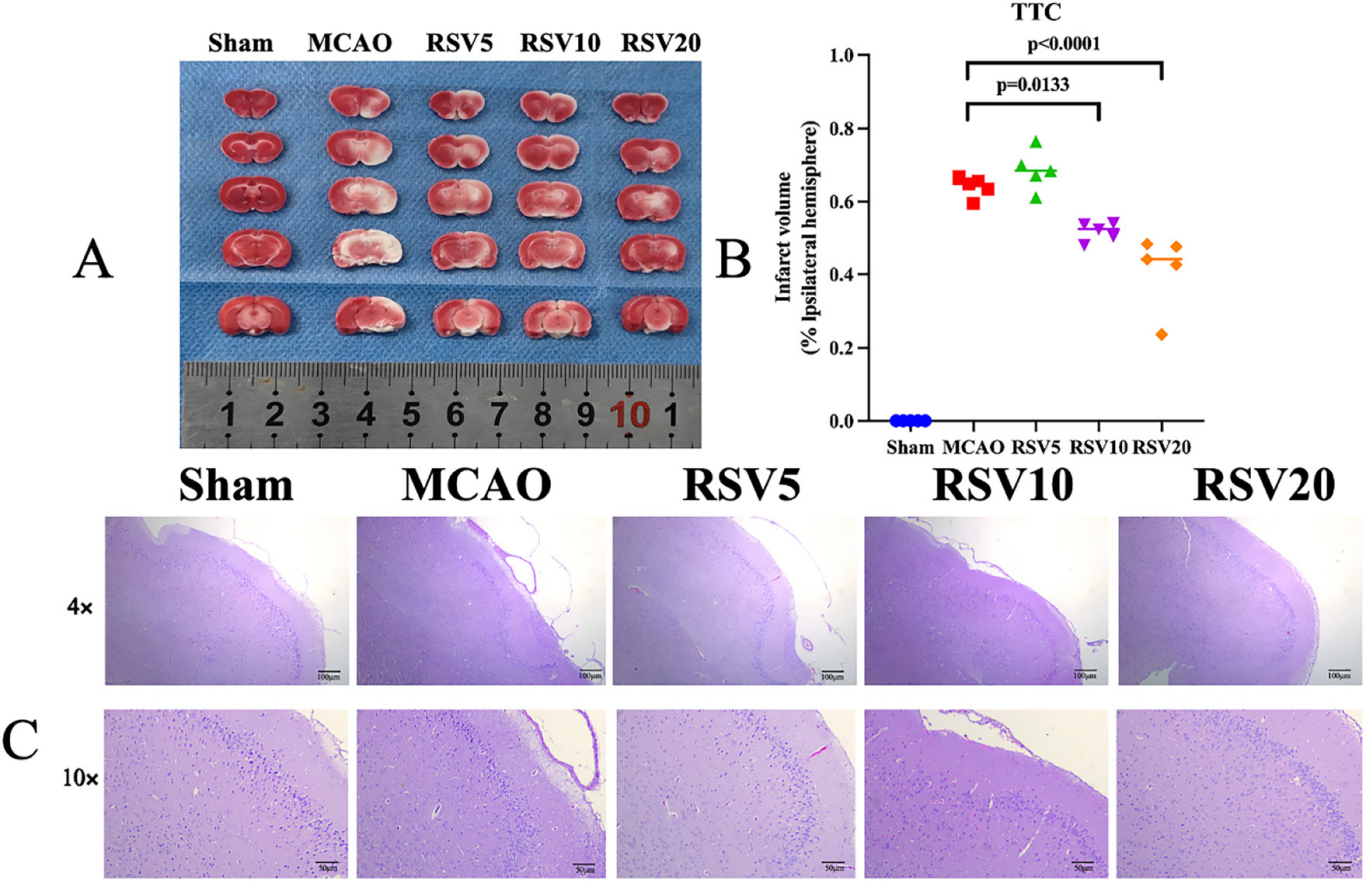
Comparison of cerebral infarct area and neuronal morphology expression in MCAO rats across groups. (A and B) Analysis and statistical results of TTC staining for cerebral infarction area in each group. In panel A, red indicates surviving neurons, whereas white indicates neuronal death. Panel B shows the statistical analysis of cerebral infarction areas in each group. (C). Hematoxylin and eosin staining of brain tissues in each group. In the sham operation group, neurons exhibited intact structures, orderly arrangement, abundant neurotransmitters in the cytoplasm, and normal synaptic gaps. In the model group, neuronal nuclei were shrunken, cytoplasmic contents were reduced, and cell alignment was disorganized.

The TTC staining results revealed that RSV significantly reduced MCAO-induced brain tissue damage. The statistical analysis of the cerebral infarction area revealed a significant difference between the RSV10 and MCAO groups (*P* = 0.0133), and an extremely significant difference between the RSV20 and MCAO groups (*P* < 0.001). The area of brain damage caused by ischemia gradually decreased with an increase in the concentration of RSV, and the brain tissue damage was significantly alleviated in the RSV10 and RSV20 groups compared with the MCAO group (Figure 3A and B). The hematoxylin and eosin staining results demonstrated that the neuronal structure in the MCAO group was disrupted, with disordered neuronal morphology. After RSV intervention, the neuronal morphology was significantly improved, and the degree of improvement gradually increased at higher RSV doses (Figure 3C). These results indicated a neuroprotective effect of RSV on the MCAO group rats, which was enhanced with increasing doses.

### 4.4 Upregulation of BDNF expression and downregulation of FABP5 and NSE expression in the brain tissue of rats with MCAO by RSV

**Figure 4:**
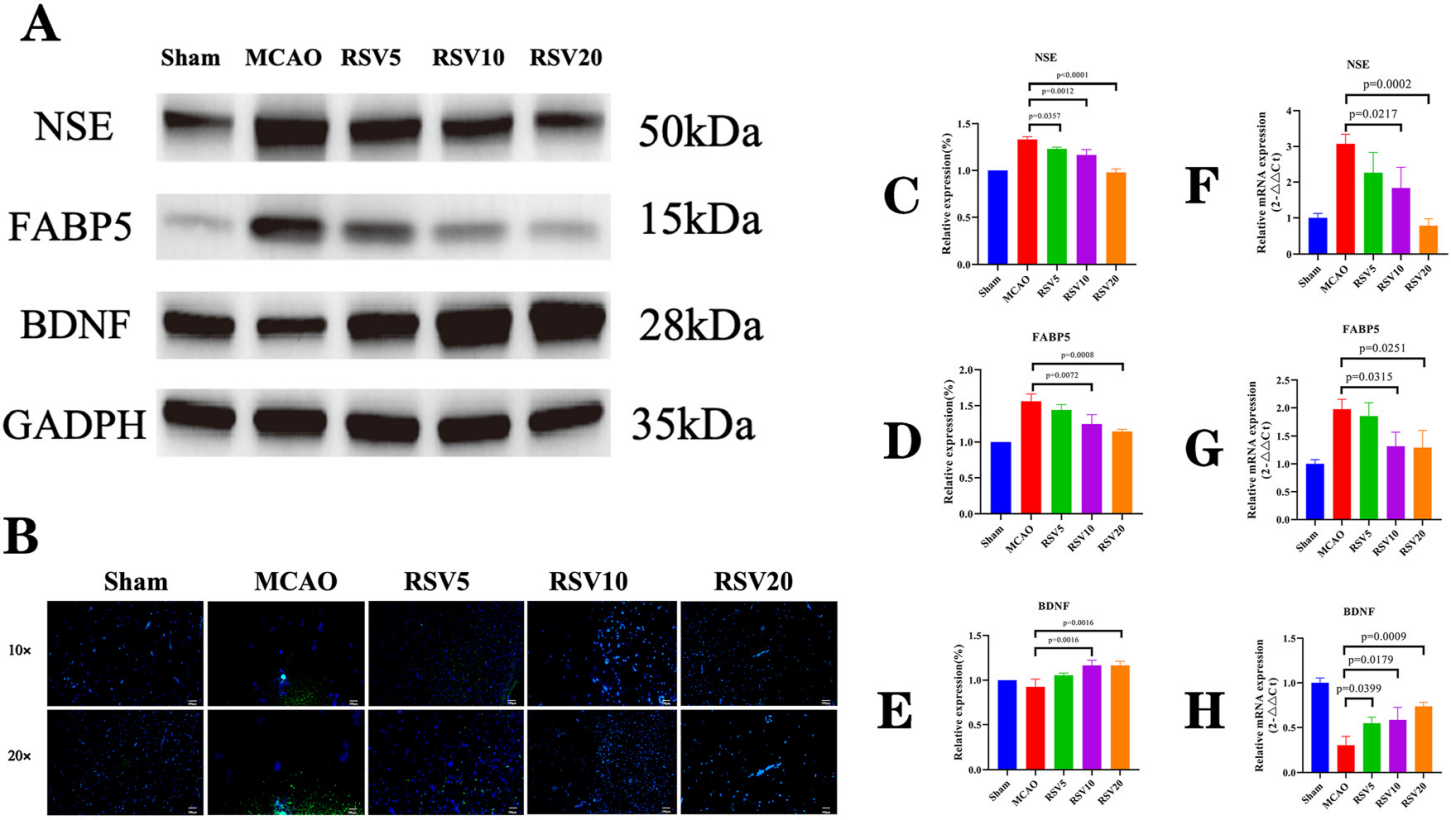
Comparison of BDNF, NSE, and FABP5 levels in brain tissues of rats across groups. (A) Western blot analysis of BDNF, NSE, and FABP5 protein expression in the brain tissues of each group. (B) Immunofluorescence detection of FABP5 in brain tissues of each group. Green fluorescence indicates FABP5 protein expression. (C–E) Western blot and relative quantification of NSE, FABP5, and BDNF expression levels in each group. (F–H) Statistical analysis of mRNA levels of NSE, FABP5, and BDNF in each group.

The WB results showed that the expression of NSE and FABP5 was significantly upregulated in the MCAO-group rats compared with the sham-group rats. NSE expression was downregulated after intervention with different doses of RSV. Significant differences were observed in the RSV5, RSV10, and RSV20 groups compared with the MCAO-group (*P* = 0.0357, *P* = 0.0012, and *P* < 0.0001, respectively) (Figure 4A and C). FABP5 expression was also significantly downregulated after RSV intervention, with significant differences in the RSV10 and RSV20 groups compared with the MCAO-group (*P* = 0.0072, *P* = 0.0008, respectively) (Figure 4A and D). Additionally, the BDNF expression was downregulated in the MCAO-group rats but significantly upregulated after RSV intervention, with significant differences in the RSV10 and RSV20 groups compared with the MCAO-group (*P* = 0.0016) (Figure 4A and E). The quantitative polymerase chain reaction (qPCR) results revealed that NSE and FABP5 expression was significantly upregulated in the MCAO-group rats. After intervention with different doses of RSV, NSE expression was downregulated, with significant differences in the RSV10 and RSV20 groups compared with the MCAO group (*P* = 0.0217, *P* = 0.0002, respectively) (Figure 4F). FABP5 expression was also significantly downregulated after RSV intervention, with significant differences in the RSV10 and RSV20 groups compared with the MCAO-group (*P* = 0.0315 and *P* = 0.0251, respectively) (Figure 4G). Furthermore, the BDNF expression was downregulated in the MCAO-group rats compared with the sham group rats but significantly upregulated after RSV intervention, with significant differences in the RSV5, RSV10, and RSV20 groups compared with the MCAO-group (*P* = 0.0399, *P* = 0.0179, *P* = 0.0009, respectively) (Figure 4H). The immunofluorescence results showed that the FABP5 expression was significantly upregulated in the brain tissue of MCAO-group rats compared with the sham group rats. FABP5 expression gradually decreased with increasing RSV concentrations. However, the expression of FABP3 and FABP4 showed no significant changes after RSV intervention (Figure 4B). These results indicated that the BDNF expression was downregulated in the MCAO-group rats. In contrast, NSE and FABP5 expression was upregulated. After RSV intervention, FABP5 and NSE expression was significantly downregulated and the BDNF expression was significantly upregulated.

### 4.5 Protection and repair of IS neuronal cells by RSV through downregulating FABP5 expression

**Figure 5:**
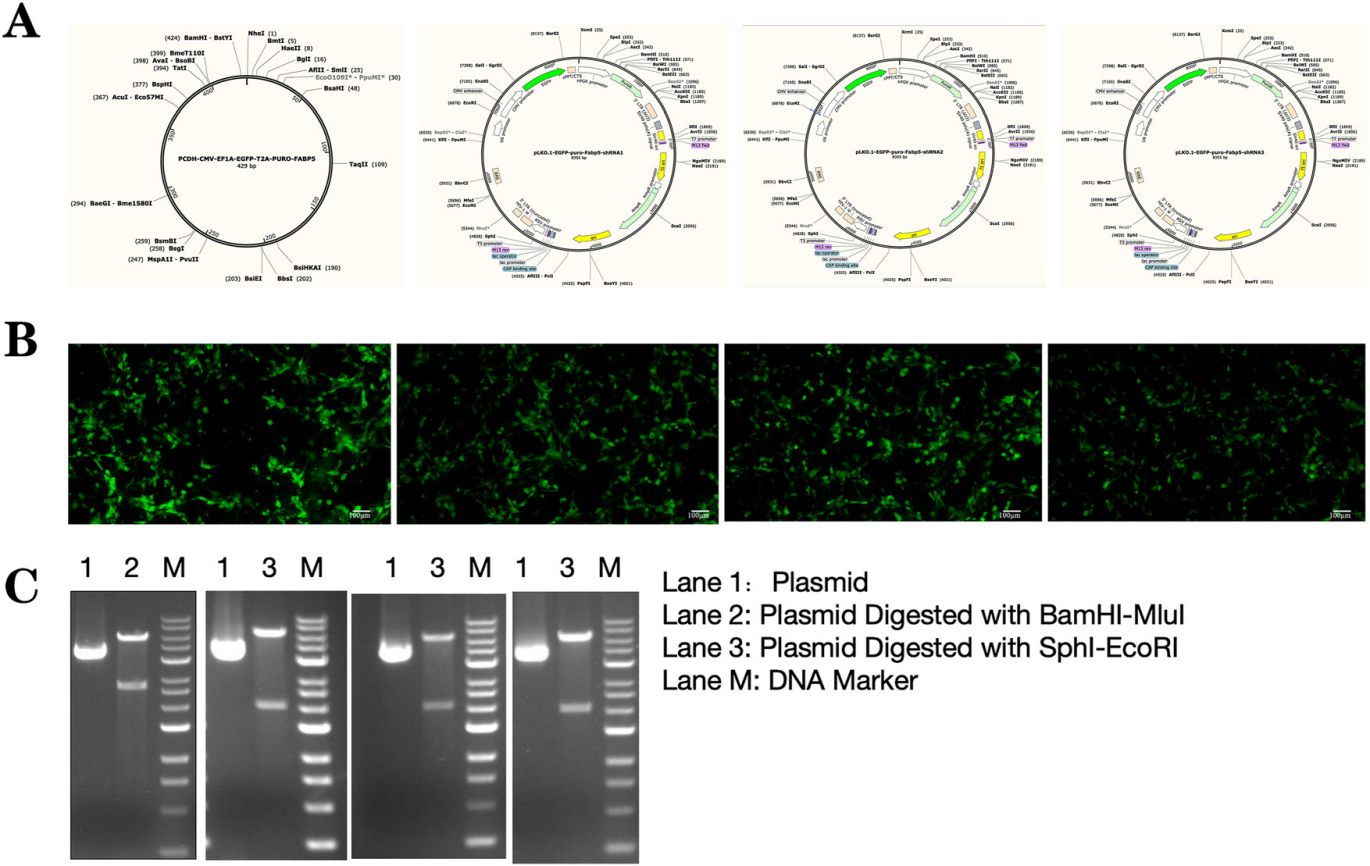
Plasmid construction and validation. (A) Schematic diagrams of overexpression and knockdown plasmids. (B) Transfection status of each plasmid in 293T cells. (C) Validation of plasmid sizes using qPCR.

**Figure 6:**
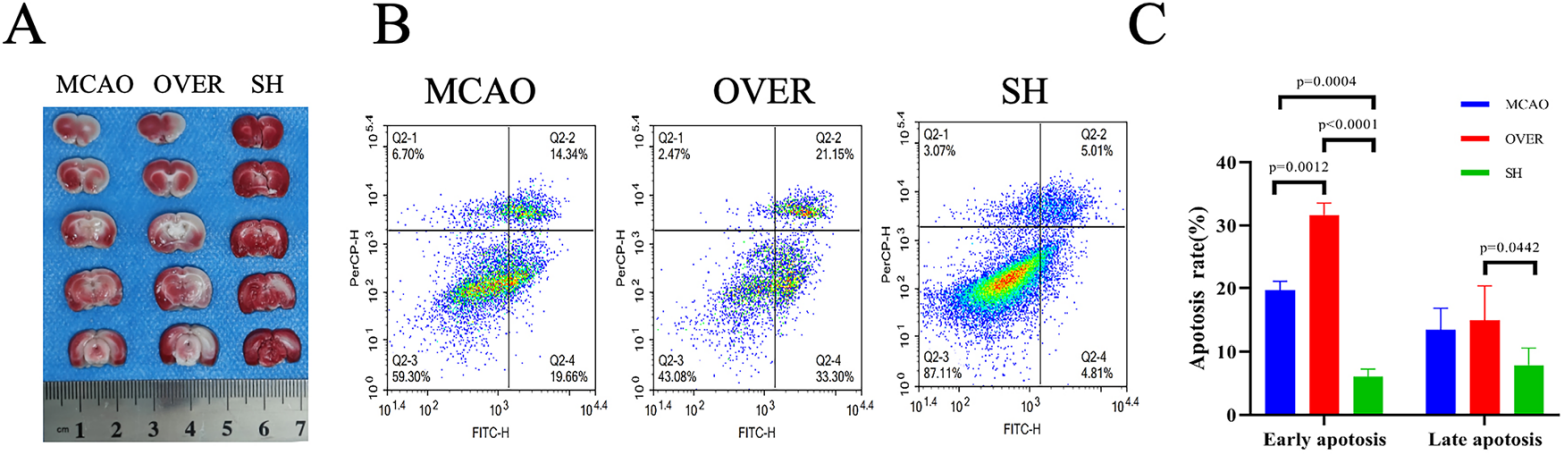
Staining results and comparison of brain tissues in the FABP5 OVER group and FABP5 SH group. (A) TTC staining of brain tissues in each group. Overexpression of FABP5 increased brain tissue damage, whereas its knockdown reversed this trend. (B) Early and late apoptosis rates of brain cells in each group. (C) Statistical analysis of early and late apoptosis rates of brain cells.

In the second part of the experiment, the FABP5 OVER group and the FABP5 SH group were subjected to FABP5 overexpression and inhibition treatments, respectively (Figure 5). Subsequently, MCAO modeling was performed, followed by RSV 10 mg/kg intervention. The results from brain tissue ischemia and flow cytometry showed that neuronal damage was significantly more severe in the OVER group. In contrast, neuronal damage was markedly improved in the SH group (Figure 6A and B). Samples were taken from the three groups after RSV 10 mg/kg intervention, and the apoptosis rates of brain neuronal cells were analyzed (Figure 6C). The early neuronal apoptosis rate significantly increased in the OVER group (*P* = 0.0012), whereas it was significantly reduced in the SH group (*P* = 0.0004) compared with the MCAO group. Additionally, the early neuronal apoptosis rate was significantly higher in the OVER group compared with the SH group (*P* < 0.0001), and the late neuronal apoptosis rate also significantly increased in the OVER group (*P* = 0.0442). These results indicated that FABP5 overexpression exacerbated IS neuronal cell damage, whereas FABP5 inhibition exerted a protective effect on IS neuronal cells. RSV may exert its neuroprotective and reparative effects by regulating FABP5 expression.

### 4.6 Downregulation of FABP5 expression by RSV to protect brain neurons possibly through AMPK-mediated energy metabolism regulation

**Figure 7:**
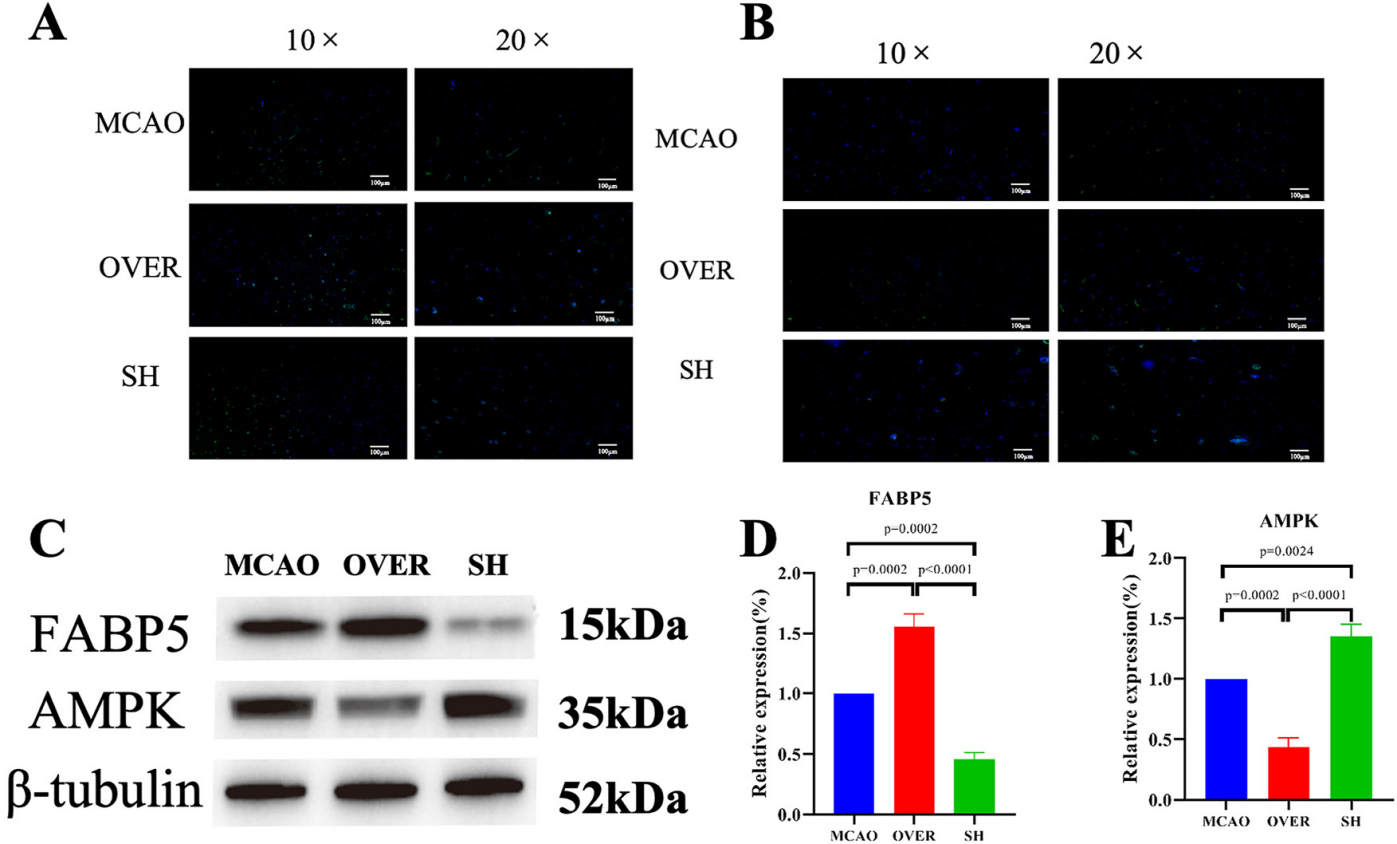
Comparison of FABP5 and AMPK expression in brain tissues between the FABP5 OVER group and FABP5 SH group. (A) Immunofluorescence staining of FABP5 in the brain tissues of rats in the three groups. Green fluorescence indicates FABP5 expression, whereas blue fluorescence indicates DAPI staining. (B) Immunofluorescence staining of AMPK in brain tissues of the three groups. Green fluorescence indicates AMPK expression, whereas blue fluorescence indicates DAPI staining. (C) Western blot bands of the brain tissues in the rats of the three groups. (D and E) RT-qPCR results showing FABP5 and AMPK expression in the brain tissues of rats in the three groups.

The immunofluorescence results showed that FABP5 expression significantly increased in the FABP5 OVER group but was significantly reduced in the FABP5 SH group (Figure 7A). Simultaneously, the AMPK expression decreased in the OVER group and increased in the SH group (Figure 7B). WB results demonstrated that AMPK expression was significantly reduced in the FABP5 OVER group but partially restored in the FABP5 SH group (Figure 7C). The reverse transcription (RT)-qPCR results revealed that FABP5 expression was significantly upregulated in the FABP5 OVER group (*P* = 0.0002) and significantly downregulated in the FABP5 SH group (*P* = 0.0002) compared with the MCAO group. Additionally, FABP5 expression was significantly higher in the OVER group compared with the SH group (*P* < 0.0001) (Figure 7D). The AMPK expression was significantly downregulated in the OVER group (*P* = 0.0002) but significantly upregulated in the SH group (*P* = 0.0024) compared with the MCAO group. Furthermore, the AMPK expression level was significantly higher in the SH group compared with the OVER group (*P* < 0.0001) (Figure 7E). These results indicated that FABP5 overexpression led to a reduction in the AMPK expression level, whereas FABP5 knockdown upregulated AMPK expression. RSV may exert its neuroprotective effects by regulating FABP5 and subsequently modulating the AMPK-mediated energy metabolism.

## 5 Discussion

The pathogenesis of IS involves multiple factors, including neuronal apoptosis, increased excitotoxicity, oxidative stress, and inflammatory responses [16], 17]. Recent studies have demonstrated that the physiological processes of stroke are driven by interactions among neurons, glial cells, vascular cells, and extracellular matrix components, all of which play crucial roles in brain tissue damage and repair [18]. The loss of neurons due to ischemia and infarction is the most direct cause of neurological functional impairment. Therefore, one of the key focuses of clinical research is to reduce neuronal apoptosis in the early stages of IS. BDNF is an essential neuroprotective factor and is often regarded as a critical indicator of neuronal repair following stroke [19], 20]. The level of NSE, a marker of neuronal damage, is significantly elevated during stroke and serves as an essential indicator for assessing neuronal injury post-stroke [21], 22]. In our study, we observed a significant increase in BDNF expression and a significant decrease in NSE expression in brain tissues after administering RSV to MCAO-group rats. These findings suggested a notable protective effect of RSV on neurons following IS.

In recent years, the influence of FA metabolism and its signaling pathways on neural repair following cerebral infarction has garnered significant attention in the scientific community. Studies have shown that certain FAs exert neuroprotective and reparative effects by binding to highly specific receptors within different lipid families [23]. Research indicates that FABPs exacerbate cerebral ischemic injury by accelerating matrix metalloproteinase-9–mediated blood–brain barrier disruption. Therefore, inhibiting FABPs is considered a potential strategy to improve outcomes in IS [24]. FABPs contribute to ischemic neuronal damage by mediating oxidative stress in neural cells, thereby influencing mitochondrial function [25]. Related studies have demonstrated a wide distribution of five subtypes of FABPs (FABP3, FABP5, FABP7, FABP8, and FABP12) in the cerebral cortex, hippocampal neuronal layers, and retinal interneurons [26], 27]. FABP3 and FABP5 have been identified as key factors contributing to mitochondrial damage in brain neurons. In this study, the analysis of FA-related pathway proteins in the brain tissue of MCAO-group rats revealed that FABP4 and FABP5 were significantly associated with neuronal repair and apoptosis following IS. However, only FABP5 levels showed significant changes after RSV administration, whereas FABP4 levels remained unaltered. Molecular docking experiments demonstrated a strong binding affinity of RSV for FABP5, suggesting that RSV may regulate FA metabolism by directly influencing the binding or transport of FAs with FABP5. In this study, we observed that FABP5 overexpression significantly exacerbated neurological functional impairment, whereas FABP5 inhibition markedly alleviated this damage. These results confirmed that FABP5 could induce CNS functional injury.

The CNS is a complex network composed of various cell types. Lipids are the second most abundant substance in the human brain after adipose tissue. Lipids primarily serve as energy reserves and utilization sources in lipid droplets, playing a crucial role in neurological diseases such as IS [28], 29]. Ischemia and hypoxia cause abnormal neuronal energy metabolism, leading to neuronal apoptosis. FA metabolism provides ATP energy to cells through β-oxidation, while also serving as a component of phospholipids, structural units of membranes, and a substrate reservoir for the synthesis of second messengers and cytokines. Additionally, FAs can induce changes in cell morphology, regulate gene expression, modulate hormone secretion, and mediate various cellular responses [30], 31]. AMPK can sense cellular energy status and regulate energy metabolism pathways through AMP activation [32]. Inhibition of the AMPK pathway can downregulate oxidative stress factors such as SOD and 4-HNE, while increasing BDNF levels to protect neurons. FABPs play a crucial role in cellular energy supply by transporting FAs, thereby influencing downstream lipid oxidative stress responses and modulating neuronal cell function [33]. By monitoring FABPs, scientists can gain insights into the energy metabolic state of corresponding organ tissues and cells. This is because FABPs play a crucial role in FA metabolism, which, in turn, can help reveal information about underlying molecular mechanisms.

The natural polyphenol RSV found in plants has been found to possess neuroprotective, cognitive-enhancing, and immunomodulatory effects. Additionally, RSV is a potent activator of AMPK [34]. *In vitro* and *in vivo* studies have shown that RSV can improve brain tissue energy metabolism and reduce energy loss by modulating the AMPK signaling pathway [35], 36]. In IS rat models, RSV exerts neuroprotective effects by inhibiting phosphodiesterase and regulating the cAMP/AMPK/SIRT1 pathway. This reduces ATP energy consumption during ischemia and significantly mitigates the detrimental effects of cerebral ischemic injury [37], 38]. In rats with IS, activating the AMPK/SIRT1/PGC-1α pathway mitigates oxidative stress, reduces mitochondrial apoptosis in brain neurons during the acute phase of cerebral ischemia, and provides a potential therapeutic basis for acute ischemic stroke [39]. In this study, RSV administration to MCAO-group rats led to improvements in open field test performance and neurobehavioral scores compared with the model group. Furthermore, RSV significantly alleviated cerebral ischemia and neuronal apoptosis in MCAO-group rats. These results suggested early RSV intervention in IS as a promising clinical treatment strategy to effectively prevent neuronal damage. In MCAO model rats with FABP5 overexpression and inhibition, we found that the AMPK expression level slightly decreased in the FABP5 SH-group but significantly decreased in the FABP5 OVER-group. This indicated that FABP5 exerted an inhibitory effect on energy metabolism in the CNS cells. The neurological function and injury in the FABP5 inhibition and OVER groups exhibited significant polarization after RSV intervention. This suggested that RSV improved brain neurological function in MCAO-group rats by inhibiting FABP5 expression and regulating brain energy metabolism pathways.

## 6 Conclusions

This study demonstrated that RSV exerted neuroprotective and reparative effects in IS, and the improvement in neurological function was significantly enhanced with an increase in the concentration of RSV. RSV ameliorated neuronal damage caused by ischemia and hypoxia by inhibiting FABP5 expression in FA metabolism and regulating brain energy metabolism pathways, thus promoting the recovery of neurological function in IS. However, this study had certain limitations. For instance, the impact of RSV – mediated by FABP5 – on other cell types (glial cells and vascular endothelial cells) remains largely unexplored. Its effects on mitochondrial metabolism and antioxidant stress responses should also be investigated in further studies.
